# Analysis of risk factors for anastomotic fistula in patients after laparoscopic radical resection for rectal cancer

**DOI:** 10.3389/fmed.2025.1705685

**Published:** 2026-01-30

**Authors:** Jun Wei, Ruijuan Jia, Lei Qiu, Haining Gan, Junxian Liang

**Affiliations:** Department of Gastrointestinal and Anorectal Surgery, The Second People’s Hospital of Foshan, Foshan, Guangdong, China

**Keywords:** anastomotic fistula, hypoalbuminemia, predictive model, rectal cancer, tumor staging

## Abstract

**Objective:**

To investigate high-risk factors for anastomotic fistula after laparoscopic radical resection of rectal cancer and establish a prediction model.

**Methods:**

This is a retrospective cohort study included a total of 306 patients diagnosed with rectal cancer who underwent laparoscopic radical resection in the Second People’s Hospital of Foshan from January 2022 to December 2024. The patients were randomly divided into a training set (*N* = 214) and a validation set (*N* = 92) in a 7:3 ratio. Models were constructed using univariate logistic regression analysis and multivariate logistic regression analysis based on the training set. Subsequently, the predictive capability of the model was evaluated using calibration curves, receiver operating characteristic (ROC) curves, decision curve analysis (DCA), and validation sets.

**Results:**

The training set included 29 patients (13.6%) who developed anastomotic fistulas postoperatively. The study identified five predictive factors: gender (*P* = 0.032, OR = 2.68, 95% CI = 1.09–6.61), tumor stage (*P* = 0.008, OR = 3.66, 95% CI = 1.41–9.49), tumor location (*P* = 0.046, OR = 2.53, 95% CI = 1.02–6.30), surgical duration (*P* = 0.031, OR = 2.72, 95% CI = 1.10–6.76), and hypoalbuminemia (*P* = 0.005, OR = 4.28, 95% CI = 1.56–11.72). The AUC of the training set is 0.776 (95% CI = 0.673–0.879). The calibration curve validation showed that the predicted and measured values were in general agreement. DCA highlighted the model’s clinical utility.

**Conclusion:**

The predictive model established in this study provides a tool for clinicians to identify high-risk patients for anastomotic fistula formation following laparoscopic radical rectal cancer surgery at an early stage. This facilitates early identification, detection, intervention and prevention of high-risk anastomotic fistula patients, thereby effectively reducing the risk of anastomotic fistula formation following laparoscopic radical rectal cancer surgery.

## 1 Introduction

Among both men and women, colorectal cancer consistently ranks as the third most common cancer, having become an ever-present threat to human health (1). In 2022 alone, approximately 1.9 million new cases of colorectal cancer were diagnosed worldwide (1). Laparoscopic radical resection for rectal cancer has been widely adopted in clinical practice due to its minimally invasive advantages, significantly improving the overall treatment outcomes for rectal cancer. However, postoperative anastomotic fistula remains a key bottleneck limiting treatment efficacy (2–6). Previous studies have demonstrated that the incidence of anastomotic fistula after laparoscopic radical resection for rectal cancer varies significantly, ranging from approximately 2.8% to 30% (7–9). Recently, scholars worldwide have extensively studied this complication, exploring its causes and influencing factors (8). Ochiai K et al. (9) discovered that when tumors are located in the mid-to-lower rectum or the anastomosis is closer to the anal margin, the risk of anastomotic fistula formation increases significantly. Furthermore, Verduin et al. (10) discovered that the risk of anastomotic fistula in patients with low rectal cancer is markedly higher than that in patients with mid - or high - rectal cancer. This may be associated with the distinctive anatomical structure and lymphatic drainage features of the low rectal area (11, 12). Although neoadjuvant chemoradiotherapy can effectively shrink the tumor size and down - stage the disease, it may induce damage to the intestinal wall tissue, impair the local blood supply, and consequently impede the healing of the anastomosis (13–15).

However, existing studies still exhibit discrepancies in the definition of risk factors. Moreover, a consensus has not yet been reached regarding some potential factors, such as the surgeon’s experience and the patient’s postoperative nutritional status. Additionally, there is a scarcity of research on the association between specific steps in laparoscopic surgery (e.g., instrument anastomosis techniques) and anastomotic fistulas (9, 15, 16). Therefore, this study aims to analyze the associated risk factors for anastomotic fistula following laparoscopic radical resection of rectal cancer through a retrospective cohort with model development/validation and to construct a risk nomogram, thereby laying the foundation for optimizing clinical prevention and treatment strategies.

## 2 Materials and methods

### 2.1 Research subjects

The study population comprised 306 patients diagnosed with rectal cancer who underwent surgical treatment in our department from January 2022 to December 2024. Among them, there were 129 male and 177 female patients. See COHORT Flow Diagram for details. All patients or their legal guardians were fully informed about the study details and provided written informed consent. The research was approved by the Ethics Committee of The Second People’s Hospital of Foshan. Number: F2021-04-20. The research presented in this manuscript adheres to the ethical norms and standards outlined in the Declaration of Helsinki.

### 2.2 Research methods

This study adopted a retrospective cohort with model development/validation and enrolled a total of 306 patients. Among these patients, 42 developed anastomotic fistula after undergoing laparoscopic radical resection for rectal cancer. The study gathered patients’ medical histories, information on smoking and alcohol consumption habits, as well as relevant data during hospitalization to explore the factors associated with the occurrence of postoperative anastomotic fistula.

### 2.3 Collecting indicators

This study gathered data regarding patients’ demographic characteristics, such as gender, age, Body mass index (BMI), comorbidities (hypertension, diabetes, coronary heart disease), lifestyle factors (smoking history, drinking history), pathological features (pathology, tumor stage, tumor location), surgical parameters (surgery time, intraoperative blood loss, anal drainage tube placement), and postoperative laboratory indicators (anemia, hypoalbuminemia, PCT levels).

Since smoking is a core risk factor for chronic obstructive pulmonary disease (COPD), and this study has already analyzed the association between smoking history and postoperative anastomotic fistula, we used smoking as an indirect measure to explore the correlation between COPD and anastomotic leakage. Consequently, COPD was not included in further investigations.

The procedure was performed by a senior attending physician in our department. Following the assessment, the primary assistant in the team provided additional support for the bowel preparation evaluation. All evaluations yielded positive results indicating adequate intestinal blood supply, with no specific data provided for determination.

Regarding issues such as diversion stoma and anastomosis technique types, both this study and the surgical team possess extensive experience in laparoscopic radical resection for rectal cancer. Consequently, the surgical approach is highly standardized. All patients enrolled in this study underwent the same surgical procedure, with virtually no differences in related surgical techniques.

### 2.4 Inclusion and exclusion criteria

#### 2.4.1 Inclusion criteria

(1) Patients diagnosed with colorectal cancer in accordance with the criteria specified in the Chinese Protocol of Diagnosis and Treatment of Colorectal Cancer (2023 edition) and who subsequently underwent laparoscopic radical resection for colorectal cancer (17);

(2) The surgical procedures were successfully completed, with all patients receiving standardized surgical interventions;

(3) Patients were aged 18 years or older, had a clear understanding of their medical condition, and possessed normal language - based communication and interaction capabilities;

(4) Patients voluntarily agreed to participate in the study and signed an informed consent form.

(5) The patient’s clinical data is complete.

#### 2.4.2 Exclusion criteria

(1) Patients with concurrent malignant tumors other than colorectal cancer;

(2) Patients presenting with severe comorbidities, including but not limited to heart, brain, kidney, or lung diseases;

(3) Patients with a documented history of mental illness or severe psychological or cognitive impairment;

(4) Patients who are non - adherent to the study protocol or explicitly refuse to participate.

(5) Missing values exist in the variables of the research subjects.

### 2.5 Missing data handling

No missing variables were observed in the enrolled patients of this study, and data collection was complete. Therefore, multiple imputation methods were not reported in this study.

### 2.6 Interpretation of relevant indicators in this study

(1) Surgical technique and peri-operative details

The lower margin of the tumor is >5 cm from the anal margin:

After general anesthesia and endotracheal intubation, position the patient in the lithotomy position. Continuously monitor vital signs (heart rate, blood pressure, blood oxygen saturation). Ensure adequate depth of anesthesia. Position adjusted based on surgical field. Laparoscopically assess the tumor’s location, size, and relationship with surrounding tissues to confirm the presence or absence of peritoneal metastasis or enlarged distant lymph nodes. Infra-mesenteric artery management: Clear lymph node No. 253 at its root. Decide whether to preserve the left colic artery based on tumor blood supply requirements to enhance anastomotic perfusion. Perform sharp dissection along the “Holy plane” to completely resect the rectal mesentery down to the levator ani muscle plane, ensuring the distal surgical margin is ≥2 cm from the lower edge of the tumor. Use a linear cutting stapler to transect the rectum 5 cm distal to the tumor. Transect the proximal colon at the splenic flexure or upper sigmoid colon, preserving sufficient intestinal length for anastomosis. The proximal colon was retracted transrectally, and a low recto-colonic anastomosis was performed using a circular stapler (28–31 mm), with the anastomotic site positioned approximately 5–8 cm from the anal margin. Methylene blue solution is injected through the anus to observe for leaks at the anastomotic site. A rectal tube may be placed for drainage if necessary. Finally, perform intra-abdominal irrigation and suture closure.

The lower margin of the tumor is ≤5 cm from the anal margin:

The anesthesia situation is the same as described above. Focus on evaluating the relationship between the tumor and the levator ani muscle and anal canal to determine whether it has invaded surrounding tissues. Routine lymph node dissection of No. 253 nodes. If the tumor is located in the anterior wall of the rectum, additional dissection of the presacral lymph nodes (No. 252) is required. Make a sharp dissection along the superior margin of the levator ani muscle to separate the rectal mesentery, preserving the anal canal epithelium and part of the internal sphincter. Use a linear cutting stapler to transect the rectum 1–2 cm distal to the tumor and transect the proximal colon at the lower sigmoid colon. Use the double - stapler technique to perform low - anterior resection while preserving the anal canal function, ensuring an adequate distal margin and proper anastomosis. An anastomosis stapler head was inserted transanally, and the colonic ends were aligned with the stapler head and anastomosed laparoscopically. Apply interrupted sutures (3-0 absorbable suture) to the anastomotic weak areas (e.g., posterior wall) to reduce the risk of fistula formation. Methylene blue solution is injected through the anus to observe for leaks at the anastomotic site. Finally, perform intra-abdominal irrigation and suture closure.

(2) Diverting stoma

The decision to place a diverting stoma is based on the actual intraoperative findings, encompassing poor patient baseline health (e.g., severe underlying diseases, advanced age), malnutrition, high anastomotic tension, surrounding tissue edema, poor anastomotic blood supply, surgical difficulties (such as anatomical anomalies, accidental injuries, prolonged operation time), and advanced tumor stage with aggressive biological behavior.

(3) Anal Drainage Tube

The placement of an anal drainage tube is mainly intended to mitigate the risk of postoperative complications in patients with rectal cancer. The decision to use it is based on the patient’s clinical condition and the surgeon’s clinical judgment. An anal drainage tube is placed when there is ultra-low anastomosis (with the anastomotic site close to the anus), reduced anastomotic healing capacity (in elderly patients, those with malnutrition, or individuals with chronic diseases such as diabetes), poor blood supply to the anastomotic site, surrounding tissue edema, or prolonged operative time.

(4) Anastomotic fistula

Definition of Anastomotic Fistula Following Laparoscopic Radical Resection for Rectal Cancer: during laparoscopic radical resection for rectal cancer, poor healing at the anastomosis may initially cause leakage of intestinal contents (anastomotic leak), which, if persistent, can lead to the formation of a pathological, epithelialized abnormal passage connecting the intestinal lumen to the abdominal cavity, other hollow organs, or the body surface (anastomotic fistula). This study strictly adopted the anastomotic fistula grading criteria established by the International Study Group of Rectal Cancer (ISGRC/ISREC) to classify the severity of anastomotic fistulas following laparoscopic rectal cancer surgery into three grades: Grade A, Grade B, and Grade C (18). Grade A perforations present no clinical symptoms and require no active therapeutic intervention. Grade B perforations necessitate active therapeutic intervention but do not require exploratory laparotomy. Grade C perforations require reoperation (18).

Diagnostic criteria:

Clinical manifestations: postoperative fever of unknown origin, especially if the temperature is ≥38 °C on the third postoperative day or if the fever persists without resolution. The abdominal drainage tube discharges cloudy, purulent, or fecal-like fluid, or emits a fecal odor. Abdominal pain and signs of peritonitis: abdominal pain, bloating, tenderness, rebound tenderness; in severe cases, diffuse peritonitis may develop. Rectal stimulation sign: anal heaviness and tenesmus; digital rectal examination may reveal anastomotic defect or fistula opening.

Laboratory examination: elevated infection markers (Increased white blood cell count, neutrophil percentage, C-reactive protein, procalcitonin, etc.); Drainage Fluid Analysis: elevated amylase levels in drainage fluid, or isolation of intestinal bacteria.

Imaging examination: CT with contrast enema: this is the key diagnostic method. CT reveals fluid or gas accumulation around the anastomosis, discontinuity of the anastomotic line, or contrast leakage.

This study collected the occurrence of anastomotic fistula within 30 days postoperatively. This study did not employ a blinded assessment method to evaluate the incidence of postoperative anastomotic fistula.

(5) Tumor Location

The classification of rectal cancer tumor location is typically determined by the distance from the lower margin of the tumor to the anal verge. Clinically, rectal cancer is commonly categorized into low - rectal cancer and mid - to - high - rectal cancer, with a 5 cm distance from the anal verge serving as the demarcation point (19). In this study, this variable was employed to explore the association between the tumor’s position relative to the anal verge and the incidence of anastomotic fistula following laparoscopic radical rectal cancer surgery.

### 2.7 Statistical methods

In this study, data processing and statistical analysis were conducted using SPSS 25.0 software. For quantitative data that followed a normal distribution, the results were presented as mean ± standard deviation (SD). Inter - group differences were assessed using independent samples *t*-tests. When the quantitative data did not conform to a normal distribution, non - parametric tests were employed for group comparisons. Qualitative data were reported as the number of cases and corresponding percentages, and the chi - square test was utilized to evaluate the presence of differences between groups. First, patients were randomly divided into a training set and a validation set in a 7:3 ratio. Perform univariate logistic regression analysis based on the training set data. Select risk factors with *P* ≤ 0.2 for inclusion in the multivariate logistic regression analysis (20). The risk nomogram model was constructed using the “rms” package in R software based on the predictors obtained from the multivariate logistic regression analysis (*P* < 0.05). After 1000 internal bootstrap validations, a ROC curve was plotted for evaluation, and a calibration curve was constructed to assess the model’s calibration accuracy. This study employed decision curve analysis to evaluate the clinical utility of the model.

## 3 Results

### 3.1 Comparison of baseline features between training set and validation set

In this experimental study, we randomly allocated the enrolled patients into a training set and a validation set in a 7:3 ratio. Specifically, the training set comprised 214 patients, while the validation set included 92 patients. General patient information from both groups was incorporated into the statistical analysis. The results of the statistical tests indicated that all *P*-values were greater than 0.05, suggesting the absence of statistically significant differences in baseline characteristics between the two groups. These findings are presented in Table 1.

**TABLE 1:** Baseline characteristic comparison.

Variables	Total (*n* = 306)	Test (*n* = 92)	Train (*n* = 214)	Statistic	*P*
Postoperative PCT	0.94 ± 0.26	0.96 ± 0.27	0.93 ± 0.26	*t* = 0.89	0.373
Group, *n* (%)				χ^2^ = 0.02	0.893
0	264 (86.27)	79 (85.87)	185 (86.45)		
1	42 (13.73)	13 (14.13)	29 (13.55)		
Gender, *n* (%)				χ^2^ = 0.49	0.482
0	177 (57.84)	56 (60.87)	121 (56.54)		
1	129 (42.16)	36 (39.13)	93 (43.46)		
Age, *n* (%)				χ^2^ = 0.15	0.702
0	178 (58.17)	52 (56.52)	126 (58.88)		
1	128 (41.83)	40 (43.48)	88 (41.12)		
BMI, *n* (%)				χ^2^ = 0.00	0.976
0	180 (58.82)	54 (58.70)	126 (58.88)		
1	126 (41.18)	38 (41.30)	88 (41.12)		
Hypertension, *n* (%)				χ^2^ = 0.07	0.795
0	213 (69.61)	65 (70.65)	148 (69.16)		
1	93 (30.39)	27 (29.35)	66 (30.84)		
Diabetes mellitus, *n* (%)				χ^2^ = 0.38	0.538
0	246 (80.39)	72 (78.26)	174 (81.31)		
1	60 (19.61)	20 (21.74)	40 (18.69)		
Coronary heart disease, *n* (%)				χ^2^ = 0.02	0.895
0	275 (89.87)	83 (90.22)	192 (89.72)		
1	31 (10.13)	9 (9.78)	22 (10.28)		
Smoking history, *n* (%)				χ^2^ = 2.31	0.129
0	258 (84.31)	82 (89.13)	176 (82.24)		
1	48 (15.69)	10 (10.87)	38 (17.76)		
Drinking history, *n* (%)				χ^2^ = 0.05	0.815
0	247 (80.72)	75 (81.52)	172 (80.37)		
1	59 (19.28)	17 (18.48)	42 (19.63)		
Pathological type, *n* (%)				χ^2^ = 0.00	1.000
0	291 (95.10)	87 (94.57)	204 (95.33)		
1	15 (4.90)	5 (5.43)	10 (4.67)		
Tumor staging, *n* (%)				χ^2^ = 0.51	0.475
0	247 (80.72)	72 (78.26)	175 (81.78)		
1	59 (19.28)	20 (21.74)	39 (18.22)		
Tumor location, *n* (%)				χ^2^ = 0.65	0.422
0	167 (54.58)	47 (51.09)	120 (56.07)		
1	139 (45.42)	45 (48.91)	94 (43.93)		
Surgery time, *n* (%)				χ^2^ = 1.30	0.255
0	191 (62.42)	53 (57.61)	138 (64.49)		
1	115 (37.58)	39 (42.39)	76 (35.51)		
Intraoperative blood loss, *n* (%)				χ^2^ = 0.25	0.615
0	183 (59.80)	57 (61.96)	126 (58.88)		
1	123 (40.20)	35 (38.04)	88 (41.12)		
Anal drainage tube, *n* (%)				χ^2^ = 0.00	1.000
0	290 (94.77)	87 (94.57)	203 (94.86)		
1	16 (5.23)	5 (5.43)	11 (5.14)		
Anemia *n* (%)				χ^2^ = 1.12	0.290
0	196 (64.05)	63 (68.48)	133 (62.15)		
1	110 (35.95)	29 (31.52)	81 (37.85)		
Hypoproteinemia, *n* (%)				χ^2^ = 4.28	0.238
0	154 (50.33)	50 (54.35)	104 (48.60)		
1	152 (49.67)	42 (45.65)	110 (51.40)		

### 3.2 Single factor analysis

A total of 214 patients from the training set were incorporated into the statistical analysis. Among these patients, 29 individuals developed anastomotic fistula subsequent to laparoscopic radical resection for rectal cancer. Potentially relevant factors were subjected to univariate logistic regression analysis. The analysis identified gender, age, smoking history, tumor stage, tumor location, surgical duration, anemia, hypoalbuminemia, and postoperative procalcitonin (PCT) levels as potential risk factors for anastomotic leakage in patients undergoing laparoscopic radical resection for rectal cancer, with all associated *P*-values being less than 0.2. Detailed information is presented in Table 2.

**TABLE 2:** Single-factor logistic regression analysis based on the training set.

Variables	β	S.E	Z	*P*	OR (95% CI)
**Gender**					
0					1.00 (Reference)
1	1.07	0.44	2.44	**0.015**	2.91 (1.23 ∼ 6.87)
**Age**					
0					1.00 (Reference)
1	0.77	0.43	1.79	0.073	2.16 (0.93 ∼ 5.01)
**BMI**					
0					1.00 (Reference)
1	0.10	0.42	0.24	0.814	1.10 (0.48 ∼ 2.54)
**Hypertension**					
0					1.00 (Reference)
1	−0.58	0.52	−1.11	0.269	0.56 (0.20 ∼ 1.56)
**Diabetes mellitus**					
0					1.00 (Reference)
1	0.38	0.50	0.75	0.451	1.46 (0.54 ∼ 3.93)
**Coronary heart disease**					
0					1.00 (Reference)
1	−0.46	0.77	−0.60	0.547	0.63 (0.14 ∼ 2.84)
**History of smoking**					
0					1.00 (Reference)
1	0.79	0.49	1.61	0.107	2.20 (0.84 ∼ 5.72)
**Drinking history**					
0					1.00 (Reference)
1	0.24	0.50	0.47	0.637	1.27 (0.47 ∼ 3.38)
**Pathological type**					
0					1.00 (Reference)
1	0.39	0.80	0.49	0.624	1.48 (0.31 ∼ 7.18)
**Tumor staging**					
0					1.00 (Reference)
1	1.77	0.45	3.93	**0.001**	5.86 (2.42 ∼ 14.15)
**Tumor location**					
0					1.00 (Reference)
1	1.78	0.52	3.43	**0.001**	5.92 (2.14 ∼ 16.38)
**Surgery time**					
0					1.00 (Reference)
1	0.88	0.42	2.07	**0.039**	2.41 (1.05 ∼ 5.54)
**Intraoperative blood loss**					
0					1.00 (Reference)
1	0.05	0.43	0.12	0.903	1.05 (0.45 ∼ 2.45)
**Anal drainage tube**					
0					1.00 (Reference)
1	−0.23	1.08	−0.21	0.832	0.80 (0.10 ∼ 6.55)
**Anemia**					
0					1.00 (Reference)
1	0.64	0.42	1.51	0.130	1.89 (0.83 ∼ 4.32)
**Hypoproteinemia**					
0					1.00 (Reference)
1	1.71	0.52	3.30	**0.001**	5.55 (2.01 ∼ 15.34)
Postoperative PCT	2.01	0.80	2.51	**0.012**	7.44 (1.56 ∼ 35.60)

The interpretation of variable assignments in this study is as follows: Group: this study categorized patients into an anastomotic fistula group and a non-anastomotic fistula group based on whether they developed an anastomotic fistula following laparoscopic radical resection for rectal cancer. Anastomotic fistula group is 1, Non-anastomotic fistula group is 0. Gender: Female is 0, Male is 1. Age: <60 years is 0, ≥60 years is 1. BMI: <24 is 0, ≥24 is 1. History of smoking and drinking: No is 0, Yes is 1. Hypertension, diabetes, coronary heart disease: No is 0, Yes is 1. Pathological type: adenocarcinoma is 0, Squamous cell carcinoma, etc., are 1. Tumor staging: assign a value of 0 to stages I–II and a value of 1 to stages III–IV. Tumor location: >5 cm from the anal margin is 0, ≤5 cm from the anal margin is 1. Surgery time: <180 min is 0, ≥180 min is 1. Intraoperative blood loss: <70 ml is 0, ≥70 ml is 1. Anal drainage tube: placement of an anal drainage tube is coded as 1, while non-placement is coded as 0. Anemia: for males, a Hb level ≥ 130 g/L is classified as normal (coded as 0 in the study), while Hb < 130 g/L is defined as anemia (coded as 1 in the study). For females, a Hb level ≥ 120 g/L is classified as normal (coded as 0 in the study), while Hb < 120 g/L is defined as anemia (coded as 1 in the study). Hypoproteinemia: hypoproteinemia was defined as serum albumin concentration < 35 g/L or serum total protein < 60 g/L (coded as 1 in the study). Normal levels were defined as serum albumin ≥ 35 g/L and serum total protein ≥ 60 g/L (coded as 0). This study assessed patients for anemia and hypoalbuminemia on the first postoperative day. Bold text indicates a *P*-value less than 0.05.

### 3.3 Multivariate analysis

The nine risk factors that were pinpointed through the univariate analysis in this study were subsequently incorporated into the multivariate analysis. The findings revealed that gender, tumor stage, tumor location, surgical duration, and hypoalbuminemia emerged as independent risk factors for the development of anastomotic fistula in patients who underwent laparoscopic radical resection for rectal cancer, with all corresponding *P*-values being less than 0.05. Detailed results are presented in Table 3.

**TABLE 3:** Multi-factor logistic regression analysis based on the training set.

Variables	β	S.E	Z	*P*	OR (95% CI)
Intercept	−4.62	0.68	−6.79	<0.001	0.01 (0.00 ∼ 0.04)
**Gender**					
0					1.00 (Reference)
1	0.99	0.46	2.14	0.032	2.68 (1.09 ∼ 6.61)
**Tumor staging**					
0					1.00 (Reference)
1	1.3	0.49	2.67	0.008	3.66 (1.41 ∼ 9.49)
**Tumor location**					
0					1.00 (Reference)
1	0.93	0.47	2	0.046	2.53 (1.02 ∼ 6.30)
**Surgery time**					
0					1.00 (Reference)
1	1	0.46	2.16	0.031	2.72 (1.10 ∼ 6.76)
**Hypoproteinemia**					
0					1.00 (Reference)
1	1.45	0.51	2.83	0.005	4.28 (1.56 ∼ 11.72)

### 3.4 Drawing a nomogram

**FIGURE 1:**
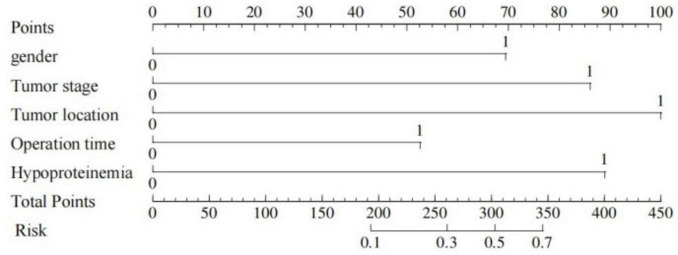
Nomogram prediction of the risk of anastomotic fistula in patients undergoing laparoscopic radical resection for rectal cancer.

**FIGURE 2:**
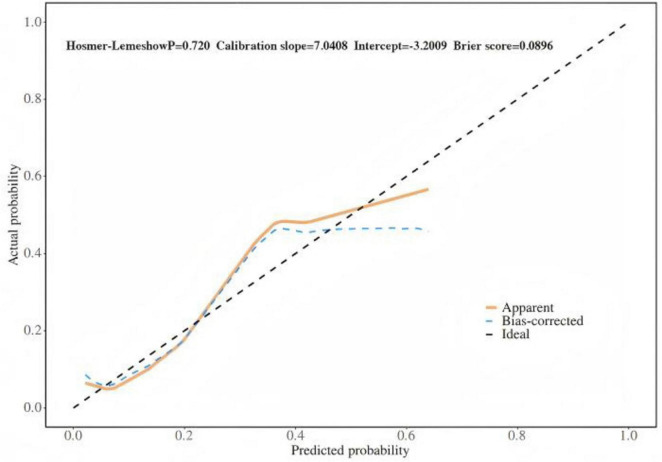
Internal validation of nomograms: calibration curves.

**FIGURE 3:**
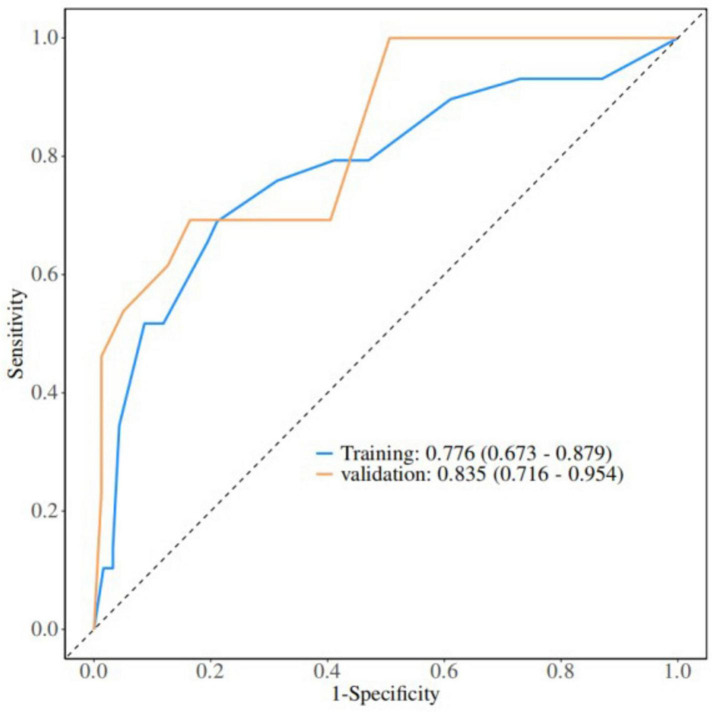
Nomogram validation: ROC. ROC, receiver operating characteristic.

**FIGURE 4:**
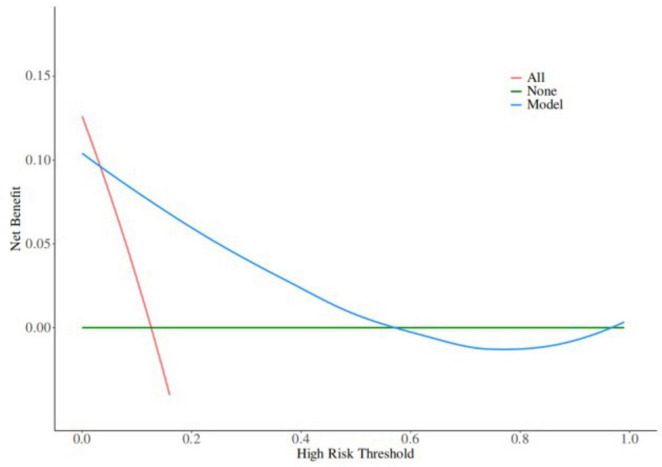
Decision curve in the nomogram model.

Based on the results of multifactor logistic regression analysis, this study constructed a nomogram for anastomotic fistula following laparoscopic radical resection of rectal cancer, see Figure 1. In applying the nomogram model, obtain variable scores from the vertical line on it first. Then sum all variable scores for a total score. Finally, determine the corresponding predicted risk value by connecting the prediction line to the total score line at the nomogram’s bottom. The calibration intercept of this study’s calibration curve is −3.2009, with a standard slope of 7.0408. This indicates that the model amplifies the variability in risk differentiation, suggesting that the actual risk for high - probability predictions may be slightly lower than the predicted values. The Brier score is 0.0896, rated as good, indicating that the current values show a low mean squared error between the model’s predicted probability and the actual occurrence of stoma closure, reflecting high predictive accuracy. The resulting calibration curve closely approximated the ideal curve, which suggests that the nomogram’s predictions regarding the incidence of anastomotic fistula in patients who have undergone laparoscopic radical surgery for rectal cancer are in strong agreement with the actual observed incidence. This high level of concordance demonstrates the nomogram’s excellent predictive performance, as depicted in Figure 2. The ROC curve of the training set of the nomogram had an AUC of 0.776 (95% CI = 0.673–0.879); the ROC curve of the validation set had an AUC of 0.835 (95% CI = 0.716–0.954), as shown in Figure 3. This finding suggests that the nomogram exhibits robust discriminatory capacity in identifying patients who are at a high risk of developing anastomotic fistula following laparoscopic radical surgery for rectal cancer. The decision - curve analysis (DCA) conducted on the nomogram reveals that when an individual’s threshold probability exceeds 0.05, the model yields greater net benefits compared to the strategies of either intervening in all patients or not intervening in any patient. This conclusion underscores the nomogram model’s significant clinical utility in predicting the occurrence of anastomotic fistula in patients undergoing laparoscopic radical rectal cancer surgery, as illustrated in Figure 4.

## 4 Discussion

In recent years, scholars both domestically and internationally have conducted research on anastomotic fistula and identified potential influencing factors. These include tumor location, especially in low - rectal cancer cases; surgical techniques, such as the proper use of staplers and accurate assessment of intestinal blood flow; and patient nutritional status, with serum albumin < 30 g/L indicating high risk (21, 22). However, owing to substantial disparities among studies with regard to sample size, as well as population characteristics that encompass confounding factors such as age, comorbidities, and regional differences, a consensus has yet to be reached among the conclusions of these studies (22, 23). In particular, the mechanisms underlying the potential risk factors, such as how glucose metabolism disorders in diabetic patients affect anastomotic healing and the inhibitory mechanism of neoadjuvant chemoradiotherapy on local tissue repair capacity, remain to be further elucidated through a combination of basic experiments and clinical research.

### 4.1 Association between gender and anastomotic fistula after laparoscopic radical resection for rectal cancer

This study’s multivariate analysis revealed that male gender is an independent risk factor for anastomotic fistula (OR = 2.68, 95% CI = 1.09–6.61, *P* = 0.032). This may be attributed to several technical limitations associated with traditional multi-port laparoscopy during mid-to-low rectal dissection, limitations that are particularly pronounced in the narrower pelvic anatomy of males. Due to objective factors such as the size of the pelvic outlet and prostate volume in males, the laparoscopic field of view and working space are both relatively limited, making dissection of the lower rectum challenging (12). The fixed position of the trocar needle limits access to the lower rectum, making it difficult to expose and retract deep pelvic structures (24). Additionally, during rectal mobilization within the narrow and deep pelvic cavity, complications such as perforation and bleeding may occur during surgery (25). Rectal perforation carries serious consequences, with a high risk of local recurrence, and the perforated site is more prone to developing fistulas compared to non-perforated areas. From a physiological mechanism perspective, higher levels of androgens in males may be a key factor (26). Androgens can inhibit fibroblast activity and the synthesis of type I collagen, which is a core structural component for anastomotic healing (26). Reduced synthesis of type I collagen directly delays the tissue repair process. At the same time, among men, the proportion of smokers and drinkers is comparatively high. Such habits can indirectly impair the healing capacity of anastomotic sites by damaging vascular endothelial function and decreasing tissue oxygen supply (23). However, current research is controversial. For example, Qi et al. did not find any gender differences (22). In clinical practice, for male patients, especially those with a history of smoking, smoking cessation education should be strengthened and nutritional status optimized before surgery. After surgery, anastomosis healing should be closely monitored.

### 4.2 The impact of tumor staging on anastomotic fistula after laparoscopic radical resection of rectal cancer

The results of this study indicate that tumor stage III–IV is an independent risk factor for anastomotic fistula (OR = 3.66, 95% CI = 1.41–9.49, *P* = 0.008). From a mechanistic perspective, advanced - stage tumors often exhibit deeper infiltration, potentially involving the vascular plexus of the intestinal wall and impairing blood supply to the anastomosis site. Furthermore, advanced - stage patients often require more extensive lymph node dissection, which may further disrupt blood supply around the intestinal tract and increase the risk of anastomotic ischemia (27, 28). This finding is consistent with the study by Nagaoka et al., which confirmed that the microvascular density at the anastomotic margin was 32% lower in patients with stage III or higher tumors than in patients with earlier stages (21). However, there is also controversy, with some scholars arguing that tumor staging is not an independent risk factor, speculating that this may be related to the higher proportion of neoadjuvant chemoradiotherapy in preoperative treatment among advanced-stage patients in this study.

### 4.3 A study on the relationship between tumor location and anastomotic fistula after laparoscopic radical resection for rectal cancer

The multivariate analysis in this study indicated that tumor location within 5 cm of the anal margin is an independent risk factor for anastomotic fistula (OR = 2.53, 95% CI = 1.02–6.30, *P* = 0.046). From an anatomical perspective, the blood supply to the rectum within 5 cm of the anal margin primarily originates from branches of the inferior rectal artery, which have relatively narrow vessel diameters and fewer anastomotic branches (19). Following surgical resection, the blood supply to the anastomotic site is prone to being affected; additionally, the narrow space around the lower rectum makes it hard to dissect the mesentery, and lymph node dissection is more likely to damage the intestinal wall vascular network, leading to reduced healing capacity of the anastomosis site. From a surgical technique perspective, low-position anastomosis must be performed within the confined pelvic cavity, where instrument manipulation may be limited by angle constraints, leading to uneven distribution of anastomotic staples and increased risk of fistula formation.

### 4.4 The mechanism by which prolonged surgical duration increases the risk of anastomotic fistula after laparoscopic radical resection for rectal cancer

The results of this study indicate that surgical duration ≥180 min is an independent risk factor for anastomotic fistula (OR = 2.72, 95% CI = 1.10–6.76, *P* = 0.031). From a mechanistic perspective, prolonged surgical duration disrupts the intra-abdominal environment. Sustained pneumoperitoneum pressure (typically maintained at 12–15 mmHg) affects intestinal blood supply, particularly microvascular perfusion in the anastomotic region. As time progresses, tissue ischemia and hypoxia worsen, leading to the accumulation of anaerobic metabolic byproducts, which directly inhibit fibroblast activity and collagen synthesis (29, 30); this is consistent with the conclusions of Kawada et al. (31). During the procedure, it was observed that after more than 3 h of surgery, the tissue oxygen partial pressure at the anastomosis site decreased by over 40% compared to preoperative levels.

### 4.5 The role of hypoproteinemia in the occurrence of anastomotic fistula after laparoscopic radical resection for rectal cancer

This study’s multivariate analysis revealed that hypoproteinemia is an independent risk factor for anastomotic fistula (OR = 4.28, 95% CI = 1.56–11.72, *P* = 0.005). In a hypoalbuminemic state, plasma colloid osmotic pressure decreases, leading to local tissue edema at the anastomosis site and impairing the diffusion of oxygen and nutrients (27, 32). At the same time, albumin, as an important nutritional substrate, the deficiency of it directly reduces the raw materials required for fibroblast proliferation and collagen synthesis, delays granulation tissue formation, and reduces the tensile strength of the anastomosis (27, 32). In addition, hypoproteinemia is often accompanied by decreased immune function, suppression of neutrophil and macrophage function, increased risk of anastomotic infection, and further hinders the healing process (27). However, some studies suggest that hypoproteinemia may be a marker of the patient’s overall nutritional status rather than a direct cause of the disease (33). This study, after adjusting for confounding factors such as age and tumor stage, still showed an independent association, indicating its direct impact on anastomotic healing (34). In clinical practice, serum albumin levels should be routinely tested prior to surgery. For patients with hypoalbuminemia, correction should be achieved through enteral nutritional support, albumin infusion, or other methods to raise preoperative albumin levels to above 35 g/L. Postoperative monitoring and maintenance of nutritional status are crucial for reducing the risk of anastomotic fistula.

### 4.6 Clinical value of a predictive model for anastomotic fistula after laparoscopic radical resection for rectal cancer

The anastomotic fistula prediction model constructed in this study incorporated five independent risk factors: gender, tumor stage, tumor location, surgery time, and hypoproteinemia. The weights of each factor were intuitively presented through a nomogram. The AUC of the training set reached 0.776 and that of the validation set increased to 0.835. The calibration curve was close to the ideal curve (Hosmer - Lemeshow *P* = 0.720). Decision curves showed that when the threshold probability was >0.05, the net benefit was significantly superior to the “full intervention” or “no intervention” strategies, demonstrating good discrimination and calibration. Compared with similar models at home and abroad, the advantages of this model are as follows: first, it focuses on the characteristics of laparoscopic surgery and excludes factors related to open surgery (such as incision length), making it more suitable for minimally invasive clinical scenarios; second, it includes tumor location (distance from the anal margin ≤ 5 cm) as a high - risk indicator specific to low - position anastomosis in laparoscopic surgery, while most models only include tumor location in general terms; third, it has been validated internally to confirm its stability, and the improvement in AUC in the validation set indicates that the model has good generalization ability across different samples. In clinical practice, it can be used for preoperative assessment (e.g., developing a stoma plan in advance for male patients with low-grade tumors and hypoalbuminemia), intraoperative decision-making (e.g., reinforcing anastomotic sites when surgery exceeds 180 min), and postoperative monitoring (e.g., increasing the frequency of imaging examinations for high-risk patients). By quantifying risks, it enables individualized interventions, thereby reducing the incidence of anastomotic fistulas.

### 4.7 Limitations of this study and future research directions

This study has certain limitations: first, as a single - center retrospective study, the 306 patients included may have been influenced by regional factors and diagnostic and treatment practices, resulting in limited sample representativeness. Additionally, the retrospective design makes it difficult to completely avoid selection bias, such as reliance on the completeness of medical records for the documentation of exposure factors like smoking and drinking history. This study did not collect data on COPD, bypass stoma, anastomosis technique type, perfusion assessment, and other factors, and thus inevitably suffers from the influence of certain confounding variables. Therefore, this study is subject to confounding factors. For some continuous variables, this study treated them as dichotomous variables, which may result in some loss of information. In addition, although the stability of the model has been confirmed through internal validation, there is a lack of multi-center external validation data, and its generalizability needs to be further tested. Based on this, future studies could include multi-center prospective cohort studies to expand the sample size and strictly control confounding factors, while incorporating more in-depth biomarkers such as intestinal barrier function indicators and inflammatory factor profiles to explore their association with anastomotic fistula formation. The weights of various risk factors in the model can also be optimized, combined with real-time monitoring indicators during surgery (such as anastomotic tension and blood supply assessment) to improve predictive performance. Randomized controlled trials can be used to validate the actual effectiveness of interventions based on this model (such as preoperative nutritional intervention and intraoperative anastomosis technique adjustments), providing more accurate prevention and treatment strategies for clinical practice.

## 5 Conclusion

This study identified gender, tumor stage, tumor location, surgical duration, and hypoproteinemia as independent risk factors for anastomotic fistula occurrence following laparoscopic radical rectal cancer surgery through multifactorial analysis. The predictive model constructed using logistic regression analysis was validated via ROC curve analysis, demonstrating good discriminatory and calibration performance. This provides a scientific quantitative tool for early identification of high-risk patients in clinical practice. The application of this model can help optimize postoperative management strategies. By implementing personalized monitoring, nutritional support, and perioperative interventions for high-risk populations, the incidence of anastomotic fistula can be significantly reduced, which has important clinical implications for improving surgical safety and enhancing patients’ long-term quality of life.

## Data Availability

The original contributions presented in this study are included in this article/supplementary material, further inquiries can be directed to the corresponding author.
